# A Treatise on Medical Electricity, Theoretical and Practical; Its Use in the Treatment of Diseases

**Published:** 1860-03

**Authors:** 


ART. II.—*A Treatise on Medical Electricity, Theoretical and Practical; and its Use in the Treatment of Paralysis, Neuralgia, and other Diseases.* By J. ALTHAUS, M.D. 8vo., pp. 352. London, 1859.

THAT electricity should have been early looked to, as a means from the application of which to the treatment of disease the most important results were to be anticipated, is not at all surprising, when we consider the character of the phenomena which result from the action of this agent under the several forms in which it presents itself. The hope of finding in one or other of the modifications of electricity a therapeutical agent adequate to the cure of some if not all of the morbid states of the nervous system that had hitherto baffled the skill of the most enlightened and experienced physicians, was doubtless rendered more sanguine by the study of its so-called physiological effects—in other words, the phenomena which result from the application of the different forms of electricity to the tissues and organs of the living body in their normal state.

At the beginning of the present century, the importance of electricity in its therapeutical appliances was warmly maintained by many competent authorities, and although a majority perhaps of the medical profession have always entertained strong doubts as to the entire accuracy of the reports in favor of its efficacy as a remedial agent, still we find that during the last sixty years, down even to the present day, there have been not a few eminent practitioners who insist that its efficiency in the cure of those cases of disease to which alone its application should be restricted, is far superior to that of any other of the therapeutic means with which we are acquainted.

There is no doubt that the differences of opinion which still exist in reference to the therapeutical value of electricity, and much of the disappointment which has been experienced in its employment for the removal of certain morbid conditions of the nerves or nervous centres, have, in a great measure, originated from an entire ignorance or an oversight of the all-important fact that the degree, manner, and form in which electricity is applied to the different portions of the living organism, will have an essentially modifying influence upon the results produced.

It is very certain that the proper and successful application of electricity, in any of its forms, for the removal of disease, is by no means so simple a thing as it was supposed to be by the older medical electricians, or as would appear still to be held by those who profess to employ it as a leading agent in the treatment of certain nervous and chronic affections. It is only from a very intimate acquaintance with the entire subject of electricity, but more especially with its effects upon the several organs of the living body, in the normal state of health, in each of its forms, in various doses, and under the different modes in which it may be applied, that we can direct it aright and with any degree of certainty, so as to obtain from it, in any case, its full remedial effects.

It has been shown, beyond the possibility of doubt, that by the application of electricity to the living tissues in their normal state, certain nervous actions, or perhaps to speak with greater accuracy, certain nervoid phenomena, varying in their character, may be produced. These effects of the action of electricity upon the living organism, will differ widely according to its quantity and intensity, to the mode in which it is transmitted to the organs of the body, and to the length of time during which its action is kept up. Hence it is evident, that to enable us to derive any success from the application of electricity to the cure of disease, a series of carefully conducted and sufficiently extended observations must be undertaken, to determine with certainty the class of cases to which it is adapted, the particular stage of each case at which it is to be employed, the manner in which it is to be administered, the degree of its intensity suited to the varying circumstances of disease, the duration of its action, and the frequency of its repetition: whether it should be made to act indiscriminately upon the different tissues—skin, muscles, parenchyma, vessels and nerves—or whether its action should be limited to a single one of these parts; whether its action is to be directed solely to the diseased part or parts, or made to embrace the entire body, or an entire limb, organ, or region. Until these questions are definitely settled, the therapeutical application and value of electricity must necessarily remain subjects of uncertainty and dispute.

A sufficient number of facts have already been recorded by some of the highest authorities in our profession, to warrant the belief that when the exact sphere of its curative powers shall be more fully ascertained, and the proper time and mode of its administration settled, electricity cannot fail to become a most valuable addition to our list of remedial agents: that by it we may be able to relieve, or even to cure permanently, many morbid conditions which, if not all of them of a very painful character, or liable to lead to a speedy extinction of life, invariably cripple the free play of important organs, and limit the powers and usefulness of the individuals afflicted with them. The whole field of medical electricity is still, in great measure, an untrodden one. It is one well adapted to yield a rich harvest to such as have the necessary qualifications and opportunities for its faithful exploration.

A number of observations bearing directly upon the therapeutical value and employment of electricity in various forms of disease are to be found scattered through the medical journals—especially those of continental Europe—through many medical works, in which the subject of medical electricity is incidentally referred to, and in numerous distinct essays, of which it is the prominent theme. These contributions, notwithstanding their intrinsic value, are available to only a small extent for the instruction of the profession generally. To adapt them for this purpose it is necessary that they should be collected together, and faithfully collated, in order that their true bearing and the positive lessons they teach may be correctly appreciated.

Heretofore we have had no reliable systematic treatise on the subject of medical electricity, adapted to the use of the working members of the profession. To supply this want has been the leading object of Dr. Althaus in the preparation of the work before us. So well has he executed his task, so well adapted is the entire treatise, in its arrangement and in the cautious tone by which it is pervaded, in respect especially to all questions of a doubtful or disputed character, connected with the general subject of medical electricity, or to the question of its propriety or the manner of its application in particular cases, that we feel no disposition to enter upon a curious research after any errors the author may have committed, or any omissions with which he may be chargeable; nor do we feel more inclined to controvert either of the positions assumed by him in regard to the therapeutical appliances of electricity, under any of the circumstances of disease to which it is supposed to be adapted. The treatise of Dr. Althaus embraces a very fair abstract of all the leading facts bearing upon the important subject of medical electricity reported by others, illustrated and enforced by a series of clinical observations collected by himself. From no other source accessible to physicians generally can the same amount of positive information be acquired upon all points connected with the remedial use of the several forms of electricity.

The field to be explored in order to the definite settlement of this subject is confessedly one of vast extent. But a short time, comparatively, has elapsed since it was opened to accurate scientific investigation, and this investigation has been entered upon as yet by only a few observers competent to conduct it to a satisfactory solution. Hence, in respect to almost everything relating to the physiological and therapeutical effects of all the forms of electricity much doubt and uncertainty prevail—the entire subject is obscured by empirical statements of the most discordant character, while the attempt to realize in practice the testimony that has been presented in favor of the curative powers of electricity by many of its enthusiastic panegyrists has too often, if not generally, resulted in disappointment, from an ignorance of almost everything relating to the indications the agent is capable of fulfilling, and the rules to be observed in its application to render certain their accomplishment.

Dr. Althaus bears testimony to the good effects derivable from certain forms of electricity, when resorted to sufficiently early and in a correct and systematic manner, in a number of cases of hysterical aphonia, paralysis, neuralgia, and various other affections of the nervous system. At the same time, he is no advocate for an entire dependence being placed in all such cases on electric treatment, to the exclusion of every internal remedy. That some, perhaps, of the morbid conditions of the nervous system may be effectually controlled by electricity alone he is quite satisfied, but, in the majority of cases, he is persuaded that a simultaneous treatment by appropriate internal remedies is of the utmost importance as tending to increase the chances of success.

In the first chapter of the treatise under consideration, the various forms of electricity are described—namely, static or frictional; dynamic, including galvanism and electro-magnetism; and animal electricity, or that produced by the general metamorphosis of matter in the living body.

In the second chapter is discussed the subject of electro-physiology—the physiological effects, in other words, produced by the action of electricity upon the healthy organs and tissues of the living body, according to the form, quantity, or intensity of its application, the mode in which it is transmitted to the part acted upon, the nature of the functions of such part, and the state of its vitality.

The various apparatus employed in the application of electricity in cases of disease, are described in chapter fourth—namely, 1st. Electrization, by bath—that is, by charging the surface of the patient's body with electricity, while he is in a state of insulation—by sparks, and by the Leyden jar. 2d. Galvanization; by the voltaic pile, the batteries of Cruikshanks, Daniell, Grove, and Bunsen; by electro-puncture; as a cautery, by Middeldorpff and Ellis's instruments; as a poultice, by Récamier's method; by Pulvermacher's chains; by the electric girdle and mixture of Breton; as a moxa, after Bird's plan. 3d. Faradisation, that is, the localization of induction currents of electricity. This method of applying electricity has been so named by Dr. Duchenne, in order to connect with it the name of Faraday, by whom the important phenomena of induction currents were discovered.

In the fourth chapter a short notice is taken of electricity as a means of diagnosis. The ensuing chapter is devoted to the consideration of electro-therapeutics, or the employment of electricity for the amelioration or cure of disease, for various surgical purposes, and for the attainment of certain ends in the practice of midwifery. In this chapter is embraced an account of the researches of Fabré-Palaprat, Klenke, Hassenstein, Richardson, Augustus Waller, and the author on the employment of electricity as a means of introducing medicinal substances into the human body, on the one hand, and on the other, the observations of Poey, Sir Humphrey Davy, and Dr. Althaus on the power of electricity to remove metallic substances out of the living body; while in an appendix some remarks are offered on the subject of atmospheric electricity and the influence produced by it upon the living organism in health and disease.

The account given by Dr. Althaus of the several forms of electricity is a very full, satisfactory, and instructive one. It embraces all that is necessary to throw light upon and direct aright its therapeutical application; the whole expressed in a sufficiently concise manner, but one at the same time clear and exact.

Frictional or static electricity was applied to the cure of disease, more especially of paralytic affections, by Kratzenstein, a German physician, as early as the year 1744, and its remedial powers in these cases were assented to by Jallabert, the Abbé Sans, Mauduit, Cavalla, Sigaud de la Fond, and several others, even up to the beginning of the present century.

"After the discovery of the voltaic pile, (1800,) and especially after that of electricity by induction, (1831,) the medicinal use of frictional electricity has been more or less abandoned, and it is only in the electricity room of Guy's Hospital, under the superintendence of Drs. Golding Bird and Gull, that therapeutical experiments on a large scale have been undertaken with it. Quite recently static electricity has again found a staunch advocate in Dr. Clemens of Frankfort, who has undertaken the irksome task of curing nearly all diseases which exist, by means of electricity. But, generally speaking, frictional electricity may be said to have disappeared from medical practice. This is especially due to the circumstance that a clumsy apparatus is required for its use, that the dose of electricity to be administered cannot be exactly regulated, and that by frictional electricity we are obliged to act indiscriminately upon the different tissues, without being able to localize the stimulus in those parts which require it."

Dr. Althaus concludes that static electricity may be entirely dispensed with in medical practice, presenting as it does, many inconveniences, in no degree compensated for by any advantages to be derived from its employment. Dynamic electricity, on the contrary, appears to him to be the true medical electricity, and it is the form now used almost exclusively in the treatment of disease.

On its first introduction, the galvanic battery was extolled in the most enthusiastic terms as a remedial agent, in cases of deafness, amaurosis, aphonia, hemiplegia, rheumatism, and even in certain forms of insanity. But, as Dr. Althaus justly remarks,—

"As great as had been the enthusiasm for the newly-discovered remedial agent, was the disappointment which necessarily followed after the unbounded expectations which had been entertained had not been realized. In most of the cases treated by galvanism no success was observed; besides which, the current furnished by the voltaic pile proved to be very inconstant, the use of the battery troublesome and expensive, and by the application of two strong currents, even accidents of a serious character were produced. The confidence in the curative powers of galvanism was, therefore, totally shaken, and the voltaic pile was ranged, together with talismans, amulets, and animal magnetism, among the pharmacopœia of quackery."

In 1825, the employment of the electro-puncture, or the application of the voltaic current, through acupuncture needles inserted into the substance of the organ to be acted on, was suggested by Sarlandière, as a suitable means of administering electricity; and Magendie is reported to have succeeded in effecting by it remarkable cures of paralysis, amaurosis, and neuralgia. Electro-puncture has been proposed for the cure of aneurism, and for cauterization when such an effect is required.

"Prévost and Dumas, Bonnet, Melicher, and Bence Jones, succeeded in decomposing calculi of the human bladder by means of the continuous current, and Mr. Spencer Wells applied the current of a single pair to promote the growth of healthy granulations and the cicatrization of ulcers. The continuous current as applied to diseases of the nervous system had again gone out of use, being replaced by the induction currents, when Dr. Remak, of Berlin, endeavored once more to prove it a first-rate remedy for those complaints."

The discovery in 1831, by Faraday, of induction currents, caused a new era in the medical application of electricity. The conditions necessary in an induction apparatus suited to the treatment of disease are very fully discussed by Dr. Althaus. After a brief examination of the question, whether the apparatus employed should be a volta-electric or a magneto-electric one, and pointing out the fact that both forms are demanded in the treatment of different cases of disease, he next shows that, first, the doses of electricity administered require to be exactly measured to suit the constitution, age, and sex of the patient operated on: every apparatus, therefore, adapted for medical use must possess a means by which the intensity of the current may be easily increased or diminished.

"The apparatus must be able to furnish currents of very high tension, or no effect would be produced in certain cases of muscular atrophy and anæsthesia, especially if electricity is to be applied on spots where the epidermis is very thick, as, for instance, on the palms of the hands and soles of the feet; on the other hand, a very mild current is generally required for galvanizing the muscles of the face in paralysis of the portio dura, and for exciting the recurrent nerve in hysterical aphonia."

Secondly, a volta-electric apparatus for medical purposes must furnish both a primary or extra-current, and a secondary current—the first being induced by the action of the spirals of the thick wire upon themselves; while the second is induced in the second wire, which is long and fine. Duchenne directs especial attention to the fact that there is a difference between the physiological action of the extra-current, which he denominates current of the first order, and that of the current induced in the second wire, denominated by him current of the second order.

"According to Duchenne, the current of the first order acts chiefly on the contractile power of the muscles, while the current of the second order acts chiefly on the sentient nerves; and on the retina, when applied by means of moistened conductors to any point of the face or scalp animated by the trigeminal nerve."

Dr. Althaus shows, finally, that another most important point in the construction of an induction apparatus is a suitable contrivance to open and close the circuit of the electro-galvanic current, inasmuch as induction currents exist only on making and breaking the circuit, and not while it remains closed.

We cannot follow the author in his description of suitable induction apparatus and their several parts, nor in his account of the method of applying induction currents invented by Duchenne, and named by him Faradisation. A good instrument and its proper use, at the proper time and in the appropriate forms and conditions of disease, are essential to the successful employment of induction currents as a therapeutic agent. For a sufficiently full and accurate elucidation of these important questions we refer the reader to the work of Dr. Althaus.

The great advantage of Faradisation is, that by it the electricity may be brought to bear directly, and in "proper degree," on the diseased organ or tissue; upon the part whose morbid condition it is our desire to rectify, without endangering any healthy part or organ, or the entire nervous system. By this mode of employing electricity therapeutically, we are enabled to act directly and solely upon the skin, and by so doing to benefit the patient in many cases of anæsthesia and neuralgia; or upon the drum of the ear, for the relief of certain cases of nervous deafness; upon the mucous membrane of the nose, to recall the lost or weakened sense of smell; upon the rectum and muscles of the anus in cases of involuntary stools, and of prolapsus ani, when produced by paralysis of the sphincter and levator ani muscles; upon the bladder and pharyngeal muscles, in cases of paralysis of either of these organs; and upon the larynx in cases of aphonia from loss of power in the nerves and muscular apparatus of this part.

"Direct Faradisation of the heart and the lungs, the stomach and the liver," remarks Dr. Althaus, "is not possible; but stomach, lungs, and heart may be excited indirectly by Faradisation of the vagi, accessible to the electric stimulus through the pharynx and œsophagus. Such operations seem, however, to be more dangerous than useful, so that a conscientious physician will seldom, if ever, consent to employ them. The electric excitation of the diaphragm may easily be produced by Faradisation of the phrenic nerve, which, taking its rise from the third, fourth, and fifth cervical pairs, proceeds downward and inward in front of the scalenus anticus, before it reaches the mediastinum and the diaphragm. The phrenic nerve may be easily excited on the anterior surface of the scalenus anticus. Conductors with large surfaces, viz., sponges lodged in metallic cylinders, are held on the point alluded to, and the artificial respiration is instantly produced; the thorax is expanded and the air rushes into the lungs with considerable noise. By this means it is possible to maintain respiration even some time after death; and it may be easily conceived how important this agent may become in cases of asphyxia, whether it be produced by charcoal fumes, opium, chloroform, or by cholera. In all these cases the first indication is to induce respiration, which is often to save life."

In respect to the use of electricity as a means of diagnosis—especially as a means of distinguishing from each other the several forms of paralysis met with in practice—a subject to which attention was first specially directed by Dr. Marshall Hall—a series of cases are first presented as representatives of the three forms of paralysis resulting from brain disease; namely, that in which the muscles are flaccid, their contractility diminished, and the polarity of the nerves depressed; that in which the muscular contractility is increased, with early rigidity of the muscles, and an irritative lesion of the brain, and that, finally, in which there is no difference to be observed in respect to the contractility of the muscles between those of the healthy and the paralytic limb. Dr. Althaus remarks:—

"We have thus arrived at the result that the muscles of paralyzed limbs may present three different conditions when subjected to the action of the electric current, and that this may enable us to form, in certain cases, the diagnosis of the paralyzing lesion.

"1. If the excitability of the muscles—or rather the polarity of the motor nerves—be *increased* in the paralyzed limb, the case is one of *cerebral paralysis*, connected with an irritative lesion within the cranium.

"2. If the excitability of the muscles be nearly or totally *lost*, we have, in all probability, either *lead-palsy* or *traumatic paralysis;* but it must be kept in mind that certain hysterical and rheumatic palsies of long standing present the same peculiarity; and that it also may be found in cases of diseases of the brain and of the cord.

"3. If *paralyzed muscles respond readily to the electric current*, there is no lead in the system, nor is the connection between the motor nerves of the paralyzed muscles and the cord interrupted; but if such cases are of *long standing*, they are due to *brain disease;* and if they are of *recent standing*, they are generally instances of *hysterical*, *rheumatic*, or *spontaneous* paralysis."

In a very clear and candid examination of the subject of electro-therapeutics, Dr. Althaus has shown by the recorded observations of several distinguished practitioners of Europe, and by a series of cases treated by himself either in King's College, and St. Mary's and the Samaritan Free Hospitals, London, or in the private practice of eminent members of the profession, by whom the cases were intrusted to him for electric treatment, that electricity has been successfully employed as a remedial agent, under various circumstances, in medicine, surgery, and midwifery. That by its curative action certain paralytic and spasmodic diseases, and certain cases of hyperæsthenia and anæsthenia, have been greatly ameliorated or entirely removed. That it has been employed with beneficial results for the discussion of rheumatic callosities. It has likewise been employed, and, it is asserted, successfully, as a means of conveying medicinal substances into the human body, as well as of removing deleterious substances from the living tissues. In respect, however, to the latter effect of electricity there are good reasons for the entertainment of very strong doubt. In surgery it has been resorted to for the cure of aneurism and varices by producing the coagulation of the blood within them; for the dissolution of urinary calculi; for actual cauterization; for the treatment of ulcers, and for effecting the absorption of exudates; and in proof of its entire adaptedness for each of these purposes, facts have been adduced of a very striking character. In midwifery electricity has been shown to be useful, occasionally, as a stimulus in cases of tedious labor from uterine inertia, and in certain forms of uterine hemorrhage.

It is in cases of paralysis, however, that electricity has been found, perhaps, most efficient as a therapeutic agent. In hemorrhage of the brain, as well as in atrophy and softening of the cerebral substance, it is true, injury rather than benefit will be the result of the application of electricity—nor is there any propriety of its employment in that form of paralysis dependent upon morbid growths within the cranium. Nevertheless, in the course of paralyses, even such as were originally produced by some lesion of the brain, the application of electricity may be found decidedly beneficial.

"When," remarks Dr. Althaus, "there has been merely an effusion of blood, the symptoms are rather caused by the clot compressing and separating the cerebral substance than by destruction of brain tissue, although it is quite true that hemorrhage into the brain is almost always accompanied with a certain amount of rupture of brain fibres. In cases where the symptoms of paralysis are prinpally caused by a clot, the patient's health may be perfectly restored. At first, the fluid parts of the blood effused into the brain are absorbed, and an organized membrane, a cyst, is formed around the clot, which in the course of time is likewise absorbed. The cyst then shrinks up, and at last only a cicatrice is to be found. In a certain number of cases this process of reparation is accompanied by a gradual amelioration of the paralytic symptoms, and thus a spontaneous recovery may take place. In other instances, by the gradual shrinking of the cyst, an irritative condition is kept up in the brain, and the paralyzed muscles then assume a rigid condition. Finally, the cicatrice may have been formed, and there may be no rigidity of the muscles, while the paralysis still continues in a more or less degree. Now, when there has been an extensive laceration of cerebral substance, and when by the shrinking of the cyst rigidity of the paralyzed muscles is induced, the paralysis depends upon central injury, and an electric treatment would only tend to aggravate the symptoms. It is also evident that if symptoms of spontaneous amelioration are observed, there is no need of electricity. But if we have reason to believe that the cicatrice has been duly formed, and nevertheless the paralysis continues, the electric current may be employed with a fair chance of success, and should be localized in the paralyzed motor nerves and muscles."

Dr. Althaus includes, also, among the palsies which are scarcely ever amenable to the influence of electricity, those dependent upon a diseased condition of the spinal cord. Recent cases of this character will be aggravated, he remarks, if treated by electricity.

In answer to the question, how is it that electricity or galvanism acts beneficially in paralytic diseases, Dr. Althaus lays down three general propositions, namely:—

"*First.* The galvanic stimulus is capable of disturbing the molecular equilibrium of the motor nerves and muscles, so as to produce the state in which they are physiologically active. This disturbance, if judiciously produced, does not cause any injury, but tends to re-establish or to ameliorate the lost or impaired vitality of the motor nerves and muscles.

"*Second.* The galvanic stimulus allows the necessary alternate contraction and expansion of the muscles, without which their nutrition is generally soon seriously impaired.

"*Third.* The galvanic stimulus, by producing contractions of the muscles, and thus augmenting the chemical changes in—that is, the oxidation of—the muscular tissue, causes a more abundant supply of arterial blood to it, which is evidenced by an increase of heat and bulk in those parts which have been galvanized, and which in its turn augments the nutrition of the muscle."

In other words, electricity or galvanism acts as a remedial agent in paralysis by restoring the lost mobility to the molecules of the nerves and muscles, and by causing contraction, and a more considerable supply of arterial blood to the paralyzed organs.

Dr. Althaus lays it down as a leading proposition, covering all cases, that in the treatment of paralysis by electricity, only inductive currents should be used. These consist of great and sudden variations, and are therefore capable of exciting the vitality of the motor nerves to the highest degree.

In respect to the direction in which the induced currents should be sent through the paralyzed limbs, there is some difference of opinion among the leading authorities on the subject. Some are in favor of their being sent in the direction which the electric current travels in the human body,—this has been termed the direct current. Matteuci, on the contrary, advocates the sending of the induction current in the opposite direction—the inverse current. Both modes—the direct and the inverse current—may be employed. In many cases of paralysis the inverse current excites much stronger contraction in the muscles than the direct; hence, to Dr. Althaus it appears justifiable to prefer it in most cases. He believes, however, that the direction of the current should be changed now and then, as, by the continued passage of a current always in the same direction, the motor nerves and muscles are more fatigued than if the position of the poles be sometimes reversed.

The intensity of the currents must be adapted carefully to the circumstances of each case. A very gentle current always should be employed at first, and the intensity of the current gradually increased until, if possible, decided contraction of the paralyzed muscles is produced.

The duration of each application of electricity must be left to the judgment of the operator. Some cases require a much longer continued application of electricity at each sitting than others. Generally speaking, Dr. Althaus believes that the continuance of the electric current should not be prolonged beyond fifteen minutes each time. The number of applications required will vary, also, in different cases. Cases of hysterical paralysis not unfrequently are entirely cured by the application of electricity repeated only a few times. Dr. Althaus cured a case of hysterical aphonia, and two of amenorrhœa, by a single application. In cases of wasting palsy—Cruveilhier's atrophy—the application of electricity must be repeated a very great number of times in order to arrest the disease and improve the condition of the patient.

Among the particular forms of paralysis which, according to Dr. Althaus, are suitable for the employment of electricity, and in which its remedial powers are most favorably displayed, are enumerated cases of palsy from lesions of the brain, when attended with entire relaxation of the muscles; provided they have continued for six months, with only imperfect recovery of muscular power; local palsy of the muscles of the eye, in cases where the disease is traceable to over-exertion or to want of exertion; palsy of the facial nerve from rheumatic effusion into the cellular tissue, between the facial muscles and the exterior branches of the portio dura, when of any standing; hysterical aphonia; most of the cases of local palsies of the extremities; hysterical paralysis; rheumatic paralysis; lead palsy; the paraplegia arising from diseases of the urinary organs, recently described by M. Leroy d'Etiolles and Mr. Spencer Wells, when it persists after the removal of the cause; wasting palsy—Cruveilhier's atrophy; intestinal atony giving rise to obstinate costiveness, and often to tympanitic distention of the abdomen; paralysis of the bladder, when not the result of disease of the brain or spinal cord.

Besides these cases of local paralysis, in the treatment of which electricity in the form of induction currents has been found to be a valuable remedy—generally relieving and often removing entirely the palsy of the muscles, when judiciously employed—Dr. Althaus notices the use of electricity as a therapeutic agent in amenorrhœa. By the application of static electricity, Dr. Bird reports the cure of twenty out of twenty-four cases of suppressed menstruation; other successful cases of the use of the continued current in this affection are recorded by Drs. Westring and de Molle: while the induced current has proved successful in the restoration of menstruation in the hands of Duchenne, Schulz, Baierlacher, and the author of the treatise under review. Of course, in cases of amenorrhœa resulting from structural disease of the ovaries or uterus, there is no indication that would warrant the administration of electricity.

In cases of an arrest of lacteal secretion by mental emotion, etc. a few applications of induction currents have caused, we are assured, the milk to reappear. The application to the mammæ of the currents is to be effected by means of moistened excitors. Cases of this kind have been observed by MM. Aubert and Becquerel.

According to Dr. Althaus, spasmodic and convulsive diseases are much less amenable to electricity than paralytic affections, inasmuch as they generally result from irritative lesions of the nervous centres. If, however, the convulsions are merely local, electricity, he thinks, deserves a trial. The most rational mode of application is said to be by sending a constant continuous current, of a certain intensity, through the affected muscles, especially in the inverse direction. Duchenne recommends the application of induction currents to the antagonists of the affected muscles. Finally, electricity of high tension can be administered as a counter-irritant to the surface, a method which has been successfully resorted to, especially in cases of chorea. Thirty-seven cases of the disease, treated by electricity applied according to the last-described method, are reported by Dr. Bird; of these, thirty were completely cured. Dr. Gull has likewise added his testimony to the curative powers of electricity in chorea.

In what is termed "writer's cramp," an affection of the muscles of the right hand, brought on often by emotion, anxiety, rheumatism, over-exertion, etc., and by wounds of the radial or ulnar nerves, the effects of electricity have been spoken of favorably. In some of the cases the disease is of a spasmodic character. The fingers, and especially the thumb of the right hand, being strongly flexed upon the palm whenever the patient attempts to write. In other cases there is palsy of the short extensor and adductor of the right thumb, and of the abductor of the forefinger, in consequence of which the pen, in writing, cannot be held steadily, while the fingers slip away from it. When the case is one of a spasmodic character, Dr. Althaus states that a continuous current applied to the flexor muscles is the appropriate treatment; but when paralytic, Faradisation is to be resorted to. In five cases of the paralytic form electrically treated by him, three were completely cured and two benefited. Even in these he thinks it probable that a cure would have been effected, had the patients been willing to submit sufficiently long to the treatment.

In cases of spasmodic wry-neck, occurring especially in adults, Dr. Althaus states that electricity of high tension, employed as a counter-irritant, and induction currents methodically applied to the antagonists of the affected muscles, have resulted in the production of an amelioration or entire cure.

In respect to the treatment of tetanus by electricity, we have not a sufficient number of well-attested facts to warrant the offering of any positive opinion.

In cases of hysterical cramps and contractions, which are of frequent occurrence and always troublesome to manage, there is reason to hope for the best results from the employment of electricity.

In the various forms of local anæsthesia, such as loss of smell, amaurosis, nervous deafness, loss of taste, etc., electricity, as we have already remarked, has been recommended as a most efficient remedy. In such cases as depend upon disease of the nervous centres, and of the sentient nerves, the direct current, as Dr. Althaus correctly remarks, can be of service only after the original lesion of the brain or nerve has subsided. The chances of success, he adds, are greater when the anæsthesia is idiopathic, of rheumatic or hysterical origin, or produced by poisoning.

Speaking of the treatment of neuralgia by electricity, Dr. Althaus says:—

"If neuralgia is caused by wounds of the nerves, by inflammation, hypertrophy, or cancer of the neurilemma, electricity cannot be expected to cure this affection; the same is the case if neuralgia originates in inflammation, caries, exostosis of the bony canals through which the nerves pass, or in diseases of the nervous centres, or in morbid states of the liver, uterus, ovaries, kidneys, etc. But if the neuralgia appears to be merely a morbid exaltation of sensibility without structural changes, or if it be caused by rheumatism, the Faradic treatment may be resorted to with a fair chance of success."

It is impossible for us, with the restricted space at our command, to follow our author, even in the merely outline manner in which we have thus far presented his views in respect to the therapeutic uses of electricity, in the entire extent of the exposition he has presented of the subject, in the remaining sections of the treatise under review.

With respect to the possibility of the introduction of medicinal substances into the human body by the aid of electricity, first suggested by Fabré-Palaprat, in 1833, and reaffirmed by Dr. Klenke, in 1846, and Hassenstein, in 1853, Dr. Althaus has no faith.

By Fabré-Palaprat it is asserted that he cured a case of sarcocele of seven years' standing by causing iodine to penetrate into the tumor by the aid of galvanism; that he cured a case of ague by quinine introduced by the same means into the body; that he cured himself of "ecstatic spasms" by galvanism, and cured other patients, suffering from blindness, apoplexy, inflammation of the bowels, epilepsy, tænia, monomania, etc. Dr. Klenke also relates a great number of more or less intractable diseases cured by the galvanic application of medicinal substances. Thus, cases of struma were cured by the galvanic introduction of iodide of potassium, cases of syphilis by the galvanic application of sublimate, etc. All of these statements are as destitute of proof as they are wanting in probability. They are either totally rejected, or held as of extremely doubtful authority by the profession generally.

In the early part of 1859, Dr. Richardson, of London, proposed the employment of electricity in connection with the application of narcotics, to produce local anæsthesia during surgical operations. For this purpose, he used Pulvermacher's chains, and a solution of equal parts of tincture of aconite and chloroform. In this manner, he asserts, he produced such a degree of anæsthesia as would permit of severe operations being accomplished with little or no pain. In a series of experiments published by Dr. Waller, one month subsequently to those of Dr. Richardson, it was shown that the application of a mixture of equal parts of tincture of aconite and chloroform to the human skin for ten or fifteen minutes would, of itself, produce a loss of vascularity and nearly complete anæsthesia; a result which is, however, neither accelerated nor retarded by the use, at the same time, of electricity. The anæsthesia which follows the application of the mixture referred to is confined exclusively to the part on which the application is made, being produced evidently by its local absorption. By this absorption, Dr. Waller has shown that death may be caused in animals, and possibly, also, in infants and children. The action of the narcotic mixture, with or without the application of electricity, when applied to the healthy skin, is likewise attended by a severe local inflammation of the most obstinate character, from which dangerous complications would be liable to be introduced into surgical operations.

"In an experiment which I have performed upon myself with chloroform and galvanism," remarks Dr. Althaus, "I found that anæsthesia was produced if a sponge saturated with chloroform was held to the skin for ten minutes, and that the connection of sponges with the poles of a battery did not sensibly accelerate the action. The pain felt about an hour after the experiment was very severe; on the following day an inflammation ensued which passed into suppuration, which continued for nine days. The pain continued very severe, and occasionally prevented sleep; the cicatrices were formed slowly, and earlier on those spots where only the chloroform had been applied, while cicatrization was very tardy on the spots where chloroform and electricity had been directed. This observation is not favorable to Dr. Richardson's idea of voltaic narcotism."

In the application of electricity to the cure of aneurism, by producing coagulation of the blood contained in the aneurismal sac, Dr. Althaus directs a platinum needle, connected with the positive pole of a galvanic battery, to be thrust into the centre of the sac, and the circuit to be closed by applying a metallic plate, connected with the negative pole, to the surface of the tumor. Bunsen, Grove, or Daniell's battery of twenty pairs, feebly charged, will be sufficient. The currents should be kept up for not less than twenty minutes.

"The drawback to his operation," as Dr. Althaus observes, "is, that it causes a great deal of pain, and that inflammation of the edges of the puncture, and of the skin covering the aneurism, may follow, and even gangrene might be the result. Besides, M. Broca has drawn attention to the fact that the clot formed by galvanism is rather soft, and liable to be dissolved, as it consists of fibrin and globules, while other clots are exclusively formed of fibrin, and are therefore very hard, and tend to rapid organization.

"M. Pétrequin, of Lyons, has cured a traumatic aneurism of the temporal artery, and an aneurism which had been produced by bleeding on the arm, (1845.) Since that time other successful cases have been published; but it is true that such aneurisms easily cede to pressure or ligatures. The galvano-puncture of aneurisms is especially to be recommended in such cases, where the other methods cannot be conveniently applied, on account of the seat of the tumor.

"A very remarkable case of aneurism has been published by Mr. Eyre, in the *Lancet* for July, 1853. In this case not the continuous, but the interrupted (induced) current was beneficially applied. It was an aneurism of the external iliac artery; a pulsating tumor in the left groin of the size of a fowl's egg; the pulsation was very strong, and accompanied with a bruit which could be traced two inches above the tumor; the limb was swollen and painful. Needles connected with the poles of an induction apparatus were thrust into the sac, and a current sent through it for a certain time. Alarming signs of inflammation supervened some time afterwards; and it was not until seventeen days after the operation had been made, that the tumor felt harder, and the pulsation became fainter; while, if the continuous current had been used in the manner described above, the tumor would have become solidified before the withdrawal of the needles. In the case described by Mr. Eyre, electricity acted merely as an excitant, and the sac was closed in consequence of irritation and adhesive inflammation, but not by chemical action. As suppuration may easily supervene in such cases, the employment of induction currents for the cure of aneurisms is, generally speaking, not justifiable."

Electricity has been employed, and, it is said, with good results, by a few French, German, and English obstetricians, to originate uterine contractions in cases in which the induction of premature labor is considered advisable; to increase the expulsive efforts of the uterus in cases of tedious labor; and to produce firm uterine contraction in cases of hemorrhage from the womb. By Professors Simpson and Scanzoni, however, the use of electricity in midwifery is considered as of very little value. They believe that in those instances in which uterine action has been apparently excited by electric agency, it has been either a mere coincidence, or it has resulted from the impression made on the mind of the patient, or been produced by mechanical irritation of the uterus, or of the abdominal parietes by the electrodes. The recent experiments of Dr. Mackenzie would seem, nevertheless, to show that, in cases of placenta prævia, when the profuse hemorrhage cannot be controlled by the plug or other means usually employed, until a sufficient dilatation of the os uteri has occurred to admit of manual assistance, as well as in cases of uterine hemorrhage occurring during the early months of pregnancy, which resist all other treatment, and where a resort to manual or mechanical interference is prevented by the constricted condition of the os and cervix uteri, electricity in the form of induction currents will prove invaluable. It is very certain that the very limited number of cases in which electricity has been employed during pregnancy, parturition, or during the puerperal state, as a therapeutic agent, are insufficient to enable us to arrive at any satisfactory conclusions as to its actual effects, or the circumstances under which its application is admissible or otherwise. From the facts collected by Dr. Althaus, and presented in the treatise before us, we draw the general conclusion, that the number of cases of disease, as well as the accidents of a surgical and obstetrical character, in which the therapeutic agency of any of the forms of electricity may be invoked, with some certainty of beneficial results, are extremely limited; while the trouble, the time, the apparatus, and the extreme care necessary for its proper administration, will necessarily operate against a resort to it, even within the narrow range of cases to which it is adapted and in which we may expect it to exert remedial powers.

Dr. Althaus has very correctly pointed out the impropriety of condemning electricity as a therapeutic means, from its occasional failure to do all the good that was expected from its judicious employment in cases and under circumstances which, to all appearance, were those best suited for the exhibition of its curative powers. The same disappointment is constantly experienced in the use of every other known remedy.

In estimating the true value of electricity as one of the materia medica the important fact is to be taken into consideration that, in very many of the instances in which it has been recommended for the cure of disease, it has been only after every other remedy had been tried without success, and when the disease had been of such long standing as to afford but little hope of ultimate recovery under any plan of treatment. What beneficial results might be obtained in certain affections of the nervous system, if the electric treatment were resorted to in an earlier stage of the complaint, may, Dr. Althaus thinks, be inferred from a perusal of the chapter in the work before us, where the effects of Faradisation in a number of cases of hysterical aphonia are detailed. He shall, he remarks, be especially gratified if he succeed in inducing a more frequent recourse to the electric treatment in certain forms, also, of neuralgia, which defy all other therapeutical means, but which are wonderfully under its control.

While, however, electricity may be resorted to in many cases, and with the very best results, as an excitant of muscular power in paralytic diseases, and of sensation in anæsthesia; as a means, also, of subduing spasm, as well as of reducing morbid increase of sensibility, as in neuralgia; it must be kept constantly in mind that, inasmuch as neither paralysis, spasm, neuralgia nor anæsthesia is of itself a well-defined disease, but merely a symptom of disease, and dependent, in different cases, on lesions of the most different and, occasionally, opposite character, electricity cannot be applied to all cases indiscriminately, in which either of those symptoms is present; to do so, as was the case formerly, is calculated, in consequence of the mischievous effects that must necessarily occur when its use happens to be under improper circumstances, to bring medical electricity into disrepute and to deprive us of the good we may have just reason to expect from its proper and appropriate administration.

In the foregoing notice of the treatise of Dr. Althaus, all that we have attempted to accomplish is to indicate in general terms the nature of its contents, and the leading views in respect to the value of medical electricity, so far as this can be ascertained from the observations bearing on the subject that have been heretofore recorded. To understand correctly the true rank which electricity now occupies, and that to which it is destined to rise or fall, as its remedial powers and the field of its applicability shall hereafter be more fully and accurately investigated and determined, it is requisite that the treatise of Dr. Althaus should be thoroughly studied as the exponent of the present state of our knowledge in respect to the subject; and from this task no one can rise without instruction on a department of therapeutics as yet but little understood and appreciated by the great body of medical practitioners. From no general notice, no brief analysis, can any correct idea be formed of the obligations which the profession owe to the author, as well for the collection and arrangement of the scattered materials necessary for the elucidation of the subject of medical electricity, as for the very able, faithful, and candid manner in which he has executed the work. Recommending the treatise earnestly to the notice of our readers, we shall conclude our brief and imperfect review by presenting the following, which are its concluding sentences. They contain an important caution to all who may feel inclined to test by clinical observations the correctness of the statements made by the author.

"In conclusion, a few words may be said on the accidents which may be caused by an injudicious application of electricity. This powerful agent is not one of those remedies which, if they do no good can do no harm; but, on the contrary, it may, in the hands of an inexperienced operator, do a great deal of mischief. Thus blindness has been caused by the application of the continuous current to the face in paralysis of the portia dura; fainting, cramps, hysterical fits, and palsies have been produced by administering too powerful currents; and fresh apoplectic attacks have been induced in patients who had suffered before from hemorrhage into the brain. Accidents of this kind can only be avoided if the operator is guided by physiological knowledge and sufficient therapeutical experience. In this remedy more than in any other the mode of application has an all-important bearing upon the results, for it is not electricity that cures diseases, but the physician who may cure diseases by means of electricity."

